# The prognosis of stage IA synchronous endometrial endometrioid and ovarian carcinomas

**DOI:** 10.1007/s00404-019-05288-5

**Published:** 2019-09-14

**Authors:** Xiangbo Zhan, Lei Li, Ming Wu, Jinghe Lang

**Affiliations:** 1 Department of Obstetrics and Gynecology, Peking Union Medical College Hospital, Peking Union Medical College and Chinese Academy of Medical Science, Shuaifuyuan No. 1, Dongcheng District, Beijing, 100730 China; 2 Department of Obstetrics and Gynecology, The Sixth Affiliated Hospital of Guangzhou Medical University, Qingyuan People’s Hospital, Qingyuan, 511500 Guangdong China

**Keywords:** Endometrial carcinoma, Endometrioid subtype, Synchronous carcinomas

## Abstract

**Introduction:**

Little is known about the prevalence and prognosis of synchronous endometrial and ovarian carcinomas. This report explores the survival outcomes of synchronous stage IA endometrioid endometrial and stage IA ovarian carcinomas in a retrospective cohort study.

**Methods:**

All cases of pathological confirmed synchronous stage IA endometrial endometrioid and ovarian carcinomas from June 1, 2010, to June 1, 2017, in a teaching hospital were reviewed. Patients were followed up to February 1, 2019. Survival outcomes were compared between patients with and without synchronous carcinomas.

**Results:**

In total, 841 cases with confirmed FIGO stage IA endometrioid endometrial carcinomas were included in the study; 33 patients (3.9%) had synchronous stage IA ovarian carcinomas, including 27 (81.8%) and 6 (18.2%) cases of endometrioid and mixed endometrioid/clear cell subtypes, respectively. After a median follow-up time of 56.8 months, 829 patients (97.9%) had definitive survival outcomes. Synchronous ovarian carcinomas had no impact on disease-free, overall or cancer-specific overall survival in univariate and multivariate analyses.

**Conclusion:**

In these patients with stage IA endometrioid endometrial carcinoma, the genuine incidence of synchronous stage IA ovarian carcinoma was very low, and synchronous carcinoma had no significant effects on survival outcomes.

**Electronic supplementary material:**

The online version of this article (10.1007/s00404-019-05288-5) contains supplementary material, which is available to authorized users.

## Introduction

Endometrial cancer (EC) is the fourth most common cancer and the sixth most common cause of cancer death in the United States [1] and the ninth most common cancer and tenth most common cause of cancer death in China [2]. Each year, there are an estimated 63.4 thousand new cases and 21.8 thousand deaths due to EC in China [2]. Despite a favorable prognosis, there is insufficient evidence to recommend screening for EC in women at average risk or in those who have an increased risk due to a history of unopposed estrogen therapy, tamoxifen therapy, late menopause, nulliparity, infertility or failure to ovulate, obesity, diabetes, or hypertension [3]. As the disease is frequently symptomatic at the early stage, EC is often diagnosed at stage I [4]. Moreover, patients with EC have a high incidence of other cancers. An estimated 3–5% of patients with EC also have ovarian cancer (OC) [5]; conversely, 10% of OCs are associated with ECs [6]. Synchronous carcinomas in the ovary and endometrium are the most frequent combination (50–70%) among all synchronous female genital tract malignancies. There have been debates on the prognosis and treatment of these tumor types in terms of primary or metastatic carcinoma. In general, patients with synchronous endometrial and ovarian carcinomas have a better overall prognosis than do patients with single-organ cancer with ovarian or endometrial spread [7]. The median 5-year disease-free survival (DFS) rate is reported to be 65% for synchronous EC and OC but is less than 50% for stage IIIA EC with ovarian spread [8].

In this study, we compared survival outcomes of patients with stage I synchronous endometrioid EC and OC and patients with only stage I endometrioid EC. The effects of adjuvant therapy on survival in patients with synchronous carcinomas were also analyzed.

## Methods

### Ethical approval and study design

This was a retrospective cohort study implemented in a tertiary teaching hospital. The Institutional Review Board from the study center approved the study (No. ZS-1428). All patients provided written consent before treatment. The registration number is NCT03291275 (*clinicaltrials.gov*, registered on September 25, 2017).

All eligible patients with stage IA EC confirmed by comprehensive staging surgery were classified into two groups: Group A, with only endometrioid endometrial carcinoma; and Group B, with synchronous endometrial endometrioid and ovarian carcinomas. The primary objective was to determine the 5-year DFS and 5-year overall survival (OS) rates of the two groups. The secondary objective was to determine the impact of adjuvant treatment on the survival outcomes of these patients.

### Patient enrollment

Detailed surgical and pathological data were collected by searching and reviewing electronic medical records from June 1, 2010, to June 1, 2017, at the study center. The inclusion criteria consisted of the following: primary endometrioid EC with or without synchronous OC; comprehensive staging surgery confirming stage IA synchronous endometrial and ovarian carcinoma; and detailed clinicopathological records available to retrospectively obtain information. The clinicopathological features examined that aided in the distinction between apparent independent primary tumors versus tumor metastases were according to the pathological criteria described by Scully et al. [9] Patients were excluded if they had non-endometrioid endometrial carcinomas, deep myometrial invasion, synchronous carcinomas of sites other than the ovary, any extrauterine metastasis or only simple hysterectomy surgery.

### Interventions and follow-up

The patients consented to comprehensive staging procedures, which included hysterectomy, bilateral salpingoophorectomy, and retroperitoneal lymphadenectomy. All adjuvant therapies followed relevant contemporary guidelines and/or were at the physician’s discretion. For grade 2–3 stage IA endometrial endometrioid carcinomas, utilization of brachytherapy was discussed, though no chemotherapy was recommended. For patients with stage IA ovarian carcinomas with grade 2–3 endometrioid and clear cell subtypes, intravenous platinum-based therapy of 3–6 cycles was recommended.

All patients were followed up until February 1, 2019. Close follow-up according to our customized protocol was provided for all patients. Recurrence was validated by physical examination, imaging and/or biopsy. Sites of recurrence were divided into categories within the pelvic cavity and distant sites. Mortality was confirmed by reviewing medical records and interviews by telephone and/or email.

### Statistics

Comparisons of continuous variables were conducted with parametric methods if assumptions of normal distribution were confirmed. Nonnormally distributed variables and categorical data were compared between the two groups using nonparametric tests. Survival curves were generated using the Kaplan–Meier method, and proportional hazards models were used to estimate hazard ratios and 95% confidence intervals for the effects of synchronous carcinomas and adjuvant therapy on DFS and OS. Multivariable analysis of DFS was performed with adjustment for important baseline risk factors. Unless otherwise stated, all analyses were performed with a two-sided significance level of 0.05 and conducted with the use of the software SPSS 22.0 (SPSS, Inc., Chicago, IL, USA).

## Results

### Patients’ characteristics

**Fig. 1:**
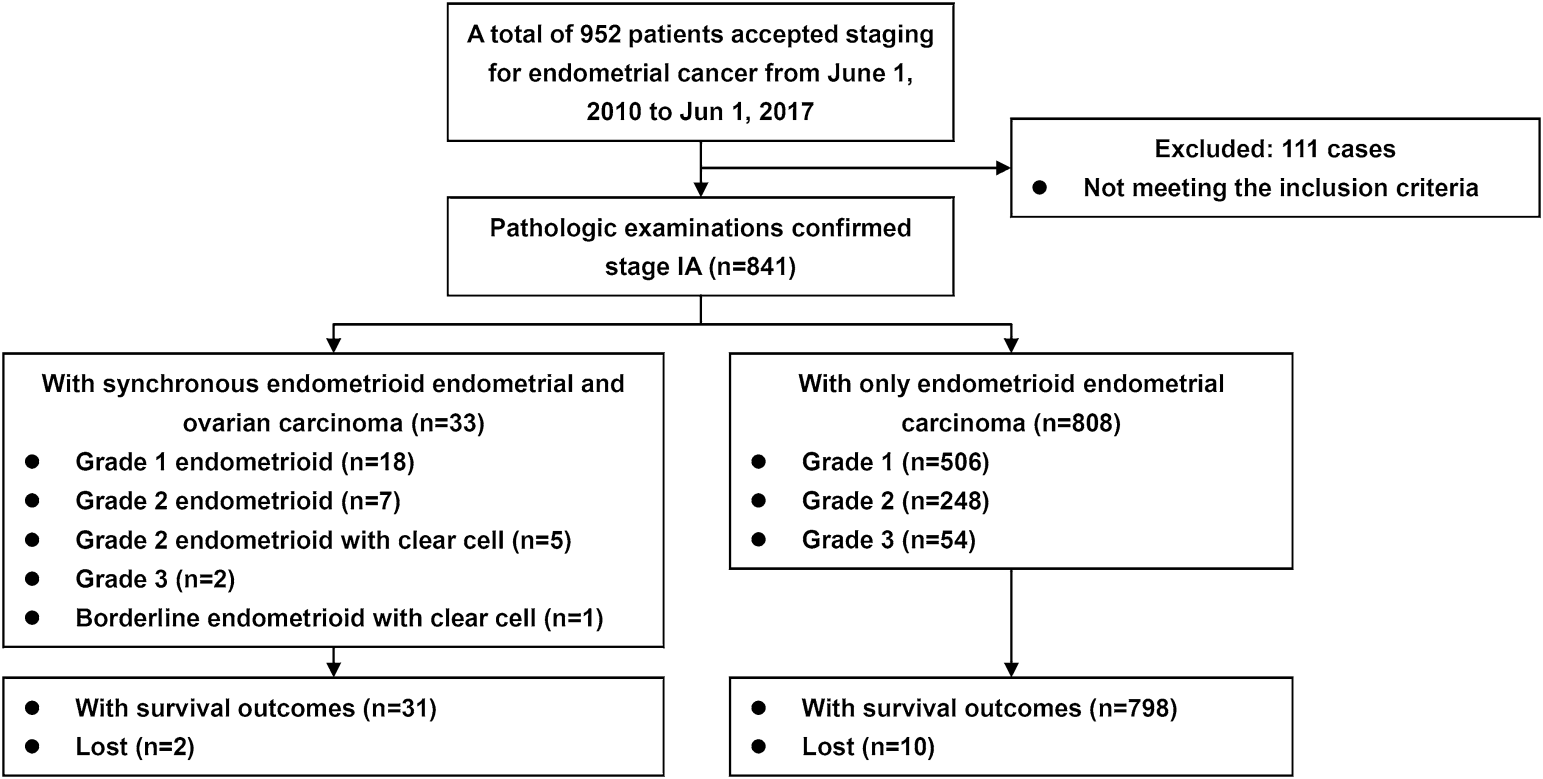
Flow diagram of the study

The patient inclusion process is shown in Fig. 1. The baseline characteristics of the patients are summarized in Table 1. From June 1, 2010, to June 1, 2017, 841 cases with confirmed FIGO stage IA endometrioid endometrial carcinomas were included in the study, involving 808 cases (96.1%) with only EC (Group A) and 33 cases (3.9%) with synchronous carcinomas (Group B). Most epidemiological and clinicopathological characteristics were well balanced (Table 1). In 33 cases of mixed carcinomas, there were 29, 3 and 1 cases of concurrent endometriosis, atypical endometriosis and borderline endometrioid in the OC specimens, respectively; there were 25 and 8 cases of atypical and complex hyperplasia in the EC specimens, respectively. The subtypes of OC were as follows: 18, 7 and 3 cases of grade 1, grade 2 and grade 3 endometrioid subtypes, respectively; 5 cases of grade 2 endometrioid with clear cell subtypes; and 1 case of borderline endometrioid with clear cell subtype.

**Table 1:** The epidemiological and clinicopathological characteristics of the patients

	Group A patients with endometrioid endometrial carcinoma (*n* = 808)	Group B patients with synchronous endometrioid endometrial and ovarian carcinomas (*n* = 33)	*p*
Age (year), mean ± SD	53.0 ± 9.1	49.5 ± 6.7	0.028
Menopause, *n* (%)	513 (63.5%)	12 (36.4%)	0.002
Metabolic disease, *n* (%)	302 (37.4%)	7 (21.2%)	0.059
Diabetes	137 (17.0%)	4 (12.1%)	0.466
Hypertension	240 (29.7%)	5 (15.2%)	0.071
Hyperlipemia	31 (3.8%)	1 (3.0%)	0.639
Obesity	30 (3.7%)	1 (3.0%)	0.654
History of infertility, *n* (%)	13 (1.6%)	2 (6.1%)	0.114
Smoking, *n* (%)	23 (2.8%)	0 (0.0%)	0.393
Diagnostic methods, *n* (%)			0.175
Dilation and curettage	417 (51.6%)	21 (63.6%)	
Hysteroscopy	391 (48.4%)	12 (36.4%)	
Surgical routs, *n* (%)			0.075
Laparoscopy	602 (74.5%)	20 (60.6%)	
Abdominal surgeries	206 (25.5%)	13 (39.4%)	
PALN resection, *n* (%)			0.151
Not done	355 (43.9%)	9 (27.3%)	
At the level of common iliac vessel	169 (20.9%)	8 (24.2%)	
Above the level of common iliac vessel	284 (35.1%)	16 (48.5%)	
Ovarian preservation, *n* (%)	14 (1.7%)	0 (0.0%)	0.568
Peritoneal cytology, *n* (%)			0.297
Not done	249 (30.8%)	13 (39.4%)	
Done	559 (69.2%)	20 (60.6%)	
Differential of endometrioid EC, *n* (%)			0.307
Grade 1	506 (62.6%)	22 (66.7%)	
Grade 2	248 (30.7%)	11 (33.3%)	
Grade 3	54 (6.7%)	0 (0.0%)	
Maximun diameter of the tumor (mm), mean ± SD	24.5 ± 19.3	26.8 ± 24.1	0.491
Tumor limited to the endometrium, *n* (%)	145 (17.9%)	10 (30.3%)	0.073
Positive LVSI, *n* (%)	54 (6.7%)	1 (3.0%)	0.350
Lower uterine involvement, *n* (%)	166 (20.5%)	8 (24.2%)	0.607
Positive peritoneal cytology, *n* (%)	18/559 (3.2%)	2/20 (10.0%)	0.149
Harvested number of PLN, mean ± SD	24.0 ± 9.7	24.6 ± 8.7	0.733
Harvested number of PALN, mean ± SD	7.7 ± 5.8	8.4 ± 4.9	0.528
Post-operative adjuvant therapy, *n* (%)	82 (10.1%)	25 (75.8%)	< 0.001
Post-operative radiotherapy, *n* (%)	62 (7.7%)	1 (3.0%)	0.275
Post-operative chemotherapy, *n* (%)	35 (4.3%)	25 (75.8%)	< 0.001
Post-operative chemotherapy protocols, *n* (%)	*n* = 35	*n* = 25	0.434
Carboplatin + paclitaxel	29 (82.9%)	22 (88.0%)	
Others	6 (17.1%)	3 (12.0%)	
Post-operative chemotherapy cycles, mean ± SD	3.6 ± 1.7	4.2 ± 1.6	0.226
Recurrent sites, *n* (%)	*n* = 19	*n* = 2	0.433
Within the pelvic cavity	7 (36.8%)	0 (0.0%)	
Distant sites	12 (63.2%)	2 (%)	

*EC* endometrial carcinoma, *LVSI* lymph-vascular space invasion, *PALN* para-aortic lymph nodes, *SD* standard deviation

### Survival outcomes of the two groups

In total, 829 patients (97.9%) had definitive survival outcomes with a median follow-up time of 56.5 months (range 17.8–105.4). There were 19 (2.4%) and 2 (6.5%) recurrences in the patients in Groups A and B, respectively; and there were 6 (0.8%) and 1 (3.2%) death, respectively. One of 7 (14.3%) deaths was not due to EC (Supplement Table 1) and occurred in Group A.

**Fig. 2:**
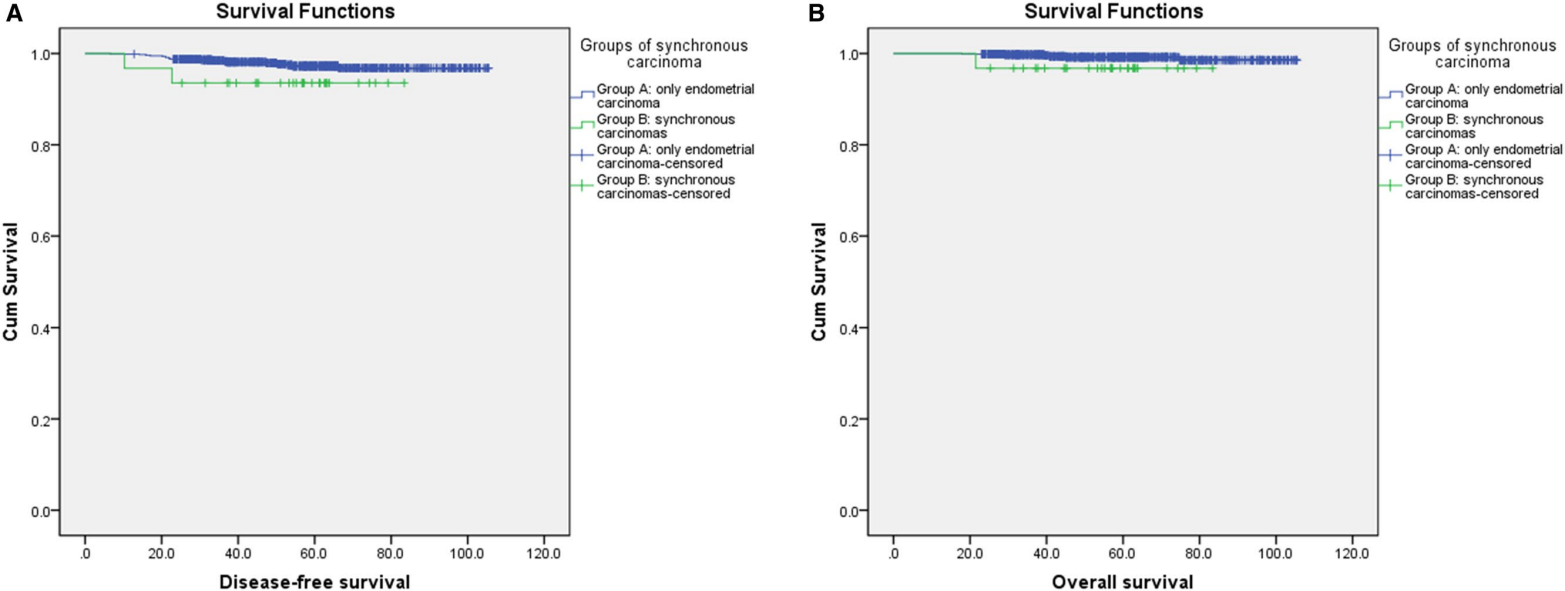
The disease-free (**a**) and overall (**b**) survival rates of the two groups

Patients in Groups A and B had similar 5-year DFS (97% and 94%, *p* = 0.172), OS (99% and 97%, *p* = 0.648) and cancer-specific OS (99% and 97%, *p* = 0.085) rates in Kaplan–Meier analysis (Fig. 2). After adjusting for baseline factors (age, menopausal status, metabolic diseases, surgical routes, tumor limited to the endometrium, postoperative adjuvant therapy), compared with Group A, the hazard ratios for recurrence, mortality and cancer-specific mortality in Group B were 1.6 (95% confidence interval: 0.2–10.0, *p* = 0.619), 11.6 (90.5–281.0, *p* = 0.130) and 8.2 (0.3–250.0, *p* = 0.225), respectively. In the Cox regression model, postoperative adjuvant therapy was the independent risk factor of DFS; no independent risk factor relevant for OS was found.

### Adjuvant treatment and survival outcomes of synchronous carcinomas

For 33 patients with synchronous carcinomas, 25 underwent adjuvant therapy, including radiotherapy in 1 case and chemotherapy in 25 cases. After a median follow-up time of 56.8 months (range 21.5–83.4), adjuvant therapy had no impact on 5-year DFS, OS or cancer-specific OS rates. No significant risk factor was found to be relevant to recurrence or mortality for patients with synchronous carcinomas.

## Discussion

In this report, we reveal that after a median follow-up of almost 5 years, patients with stage IA synchronous endometrial and ovarian carcinomas and patients with stage IA endometrial carcinomas had similar DFS and OS rates, even with adjustments for adjuvant therapy. These findings are consistent with the conclusions of a case–control study using the Surveillance, Epidemiology, and End Result Program between 1973 and 2013, which suggested that the presence of synchronous OC is not associated with EC-specific survival (10-year rates 96.0% versus 95.3%, *p* = 0.97) or OS (85.6% versus 87.2%, *p* = 0.10) [10]. Moreover, the presence of synchronous endometrioid EC does not influence the prognosis of patients with stage I OC, even in the presence of deep myometrial invasion [11]. Other authors have found that the OS rate of EC patients who had other cancers was worse than that of patients without other cancers but that disease-specific survival was not significantly different [12]. Based on these data, an appropriate prognosis could be assured within this clinical context.

In our report, all specimens of synchronous carcinomas were reviewed again to guarantee a definitive, strict diagnosis. The distinction between synchronous primary endometrial and ovarian carcinomas and metastatic malignancy is based first on histopathological criteria [9], which poses significant problems both because these criteria constitute an empiric tool based on the personal experience of the pathologist in charge and because they must account for heterogeneity among the samples analyzed [13]. A precise diagnosis is mandatory because the diagnosis is associated with a specific prognosis and needs a specific surgical and postoperative management approach, requiring extreme caution [13]. Regardless, no single criterion is perfect, and it is important to integrate all available clinicopathological, immunohistochemical and molecular data in the assessment of problematic diagnostic cases of synchronous carcinomas [14].

Whether molecular analysis can differentiate primary and metastatic carcinomas remains controversial despite several reports on synchronous carcinomas [14]. The most important limitations of these studies are the heterogeneity of tumor samples and the interpretations of findings. Synchronous gynecological carcinomas in Lynch syndrome are molecularly concordant, suggesting shared origins [15, 16]. Based on both targeted and exome sequencing, synchronous carcinomas show evidence of a clonal relationship, suggesting that among synchronous carcinomas, disseminating cells are restricted to physically accessible and microenvironment-compatible sites yet remain indolent, without the capacity for further dissemination [17]. These findings all suggest that synchronous carcinomas are not independent but rather represent an isolated metastatic phenomenon. However, this truly is a unique phenomenon, that is, patients with synchronous disease have a good prognosis following surgery, and systemic treatment need not be administered [18]. From a practical point of view, only conventional morphological criteria should be used for the classification (staging) of these tumors. Molecular profiling of these tumors may have prognostic and predictive meaning [19, 20].

In this study, adjuvant therapy had no significant positive impact on the survival outcomes of patients with synchronous carcinomas. According to current guidelines, observation can be provided for patients with grade 1 and grade 2 ovarian endometrioid carcinomas; intravenous platinum-based therapy can be provided for high-grade ovarian carcinoma or the clear cell subtype [21]. For stage IA endometrial endometrioid carcinomas, observation can be considered for patients with all grades [22]. To date, no consensus or guidelines have been achieved for the treatment of stage IA synchronous carcinomas. In a retrospective study in Japan, 2 of 23 patients with synchronous EC and OC experienced recurrence after adjuvant therapy, though none of 10 patients who did not receive adjuvant therapy had recurrence [23]. Studies on high-risk factors of recurrence may provide an index or indications for adjuvant therapy [24, 25]. Interestingly, concordant tumor grade was not associated with survival for stage I synchronous carcinomas in a large case–control study [10]. Due to the scarcity of synchronous carcinomas, a pooled analysis or meta-analysis likely provides a more substantial description of the role of adjuvant therapy in synchronous carcinomas.

In our study, most synchronous carcinoma patients (60.6%) accepted open surgeries. However, the surgical approach (open versus minimally invasive surgery) for ovarian cancer has been a topic of controversy. As reported in our study, laparoscopic staging for apparent stage I epithelial ovarian cancer appeared to be as safe as laparotomy, both in the report from National Cancer Data Base [26] and in expert opinions of the German Society for Gynecologic Endoscopy [27].

The strengths of our study are the large cohort and rigorous long-term follow-up. The limitations of our study mainly involve its retrospective design, which may introduce significant observation and selection biases. The relatively small population of synchronous carcinomas limited the analysis of adjuvant treatment in terms of survival outcomes. In our retrospective cohort, 4.3% of stage IA endometrial cancer patients and 75.8% of synchronous cancer patients received adjuvant chemotherapy. As most patients had grade 1 subtypes, the high rate of chemotherapy utilization reflects an attitude of defensive medicine and the practice of overtreatment, especially for grade 1 cervical cancer patients. A well-designed prospective study should standardize the uniform treatment protocol to overcome the abovementioned bias. A lack of molecular analysis for the various components of synchronous versus metastatic carcinomas is another shortcoming. Because synchronous endometrial and ovarian neoplasia appears to be as common in younger women without obvious EC risk as in patients with possible Lynch syndrome [28], an exploration of tools for genetic analysis is also essential for the study of synchronous carcinomas. Lastly, a longer follow-up is essential for survival analysis of patients with mixed carcinomas.

## Conclusions

In patients with stage IA endometrioid endometrial carcinoma, the appearance of synchronous stage IA ovarian carcinoma is very low and has no significant effects on survival outcomes. Adjuvant therapy does not influence the survival outcomes of this population.

## Electronic supplementary material

Below is the link to the electronic supplementary material.

Supplementary file1 (XLSX 457 kb)

## Abbreviations

DFS: Disease-free survival
EC: Endometrial cancer
OC: Ovarian cancer
OS: Overall survival
PALN: Paraortic lymph node
PLN: Pelvic lymph node
